# Return to Sport Using Corticosteroid Injections for Knee Pain in Triathletes

**DOI:** 10.7759/cureus.39985

**Published:** 2023-06-05

**Authors:** Mackenzie B Norman, Emily R Norman, Gregory H Langer, Matthew R Allen, Leo Meller, Kenneth C Vitale

**Affiliations:** 1 Orthopedic Surgery, Yale New Haven Hospital, New Haven, USA; 2 Physical Medicine and Rehabilitation, Dartmouth Geisel School of Medicine, Hanover, USA; 3 Engineering, Johns Hopkins University, Baltimore, USA; 4 Orthopedic Surgery, University of California San Diego School of Medicine, La Jolla, USA

**Keywords:** return to sport, knee joint, cortisone, athletes, triathlon

## Abstract

Introduction

Despite the prevalence of corticosteroid injections in athletes, little is known about their efficacy in triathletes. We aim to assess attitudes, use, subjective effectiveness, and time to return to sport with corticosteroid injections compared to alternative methods in triathletes with knee pain.

Methods

This is an observational study during the COVID-19 pandemic. Triathletes answered a 13-question survey posted to three triathlon-specific websites.

Results

Sixty-one triathletes responded, 97% of whom experienced knee pain at some point in their triathlete career; 63% with knee pain received a corticosteroid injection as treatment (average age 51 years old). The most popular attitude (44.3%) regarding corticosteroid injections was "tried them, with good improvement". Most found the cortisone injection helpful for two to three months (28.6%), or more than one year (28.6%); of individuals who found the injections useful for more than one year, four-eight (50%) had received multiple injections during that same period. After injection, 80.6% returned to sport within one month. The average age of people using alternative treatment methods was 39 years old; most returned to sport within one month (73.7%). Compared to alternative methods, there was an ~80% higher odds of returning to sport within one month using corticosteroid injections; however, this relationship was not significant (OR=1.786, p=0.480, 95% CI:0.448-7.09).

Conclusion

This is the first study to examine corticosteroid use in triathletes. Corticosteroid use is more common in older triathletes and results in subjective pain improvement. A strong association does not exist for a quicker return to sport using corticosteroid injections compared to alternative methods. Triathletes should be counseled on the timing of injections, duration of side effects, and be aware of potential risks.

## Introduction

Triathlon became an Olympic sport in 2000 [1] and is growing in popularity with over four million participants worldwide [2]. Chronic overuse injuries are abundant, with a 56% reported prevalence in the Ironman triathlete population [1]. In particular, lower limb injuries account for the majority of injuries sustained by triathletes, with the knee joint being the most commonly reported site of injury [3], considering its chronic overuse related to cycling and running [3,4]. Common treatment modalities for knee pain include rest, activity modification, physical therapy (PT), knee bracing and taping, non-steroidal anti-inflammatory medications (NSAIDS), and injections, including corticosteroid (cortisone) injections [4-6]. 

The anti-inflammatory effects of corticosteroids (cortisone and synthetic derivatives, commonly referred to as "cortisone") have led to common and extensive use by clinicians in the management of athletic musculoskeletal injuries [4]. Corticosteroids are commonly used for joint injections, especially in the knee [5], to treat sports-related knee pain [6]. Known risks and side effects of cortisone injections include cartilage degeneration, tendon tearing, and potential negative effects on bone mass [7]. Although cortisone injections have been extensively studied and are widely used in sports medicine in general [4], the prevalence and effectiveness of corticosteroid injections in the triathlete population are largely unknown. To the best of our knowledge, no existing literature exists on this topic. 

With the rising popularity and high rates of overuse injuries, triathletes are a key target population to study regarding cortisone injections and return to sport, as these injections are used for similar overuse injuries in the general athlete population [8]. Presently, we aim to assess attitudes regarding using corticosteroid injections for knee pain, the percentage of triathletes who utilized corticosteroid injections for knee pain, their subjective effectiveness, and the time to return to sport in triathletes using this treatment option compared to alternative methods. 

## Materials and methods

Participants 

**Figure 1:**
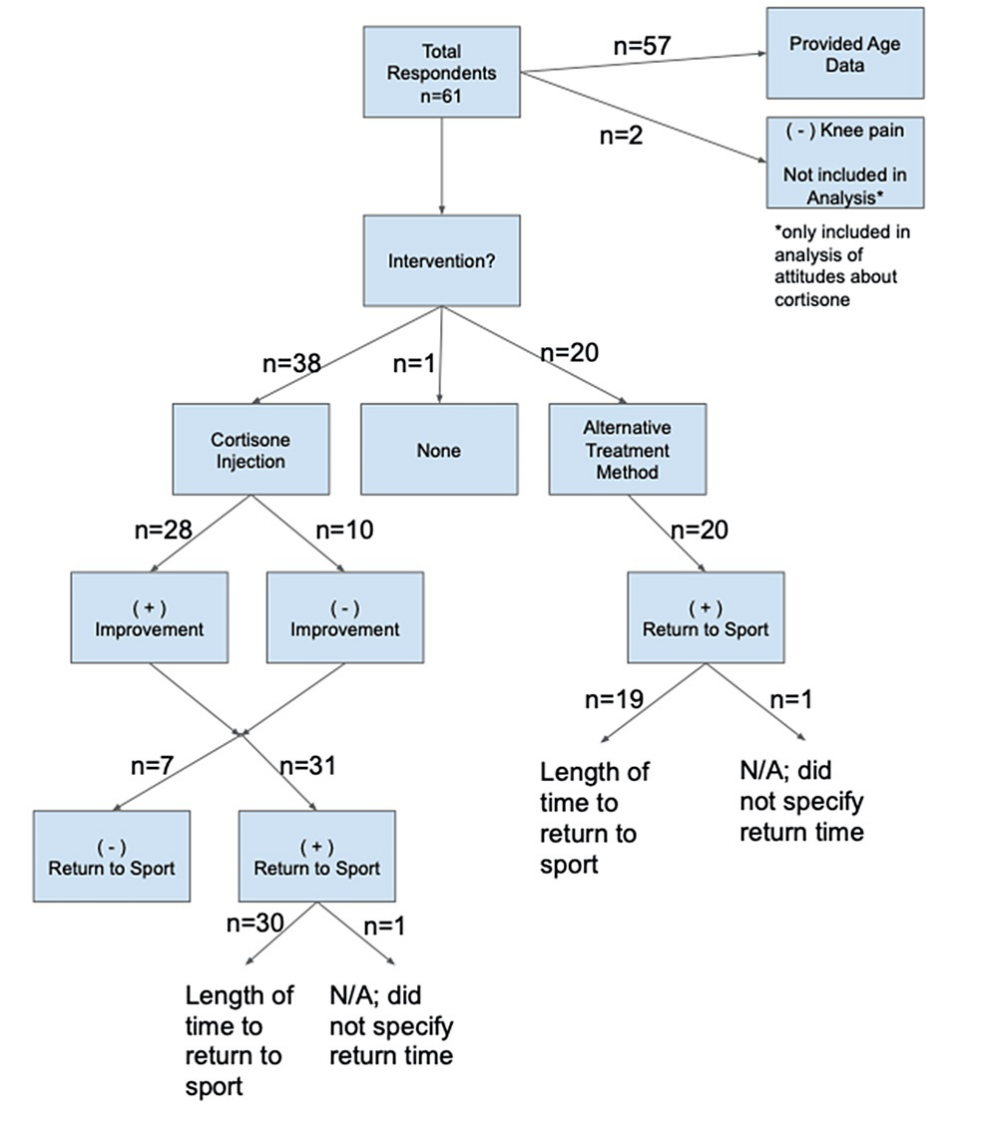
Flow diagram for inclusion and exclusion of study

This study was approved for exemption by the University of California San Diego Institutional Review Board (#200461) and all research and activities associated with this project were conducted in compliance with the institution's Human Research Protection Program (HRPP) standard operating policies and procedures. The study design was an observational study that took place during the initial phase of the COVID-19 pandemic. A 13-question electronic survey was internally distributed to test answerability and subsequently posted to social media internet platforms. The inclusion criteria was the completion of required questions in the survey by self-identifying triathletes; respondents who used both cortisone injections and alternative treatments were excluded from the analysis. We defined "alternative treatments" as non-corticosteroid injection therapies such as physical therapy (PT; weekly rehabilitation sessions with a physical therapist to improve leg strength and mobility), rest, or a knee brace. Those who did not specify age range (n=4) were not included in overall respondent demographic data but were included in the analysis of intervention type; those who did not have knee pain (n=2) were not included in the analysis of intervention type but were included in the analysis regarding attitudes towards cortisone. Figure 1 demonstrates the flow diagram of inclusion and exclusion criteria for our study. 

Protocol 

**Figure 2:**
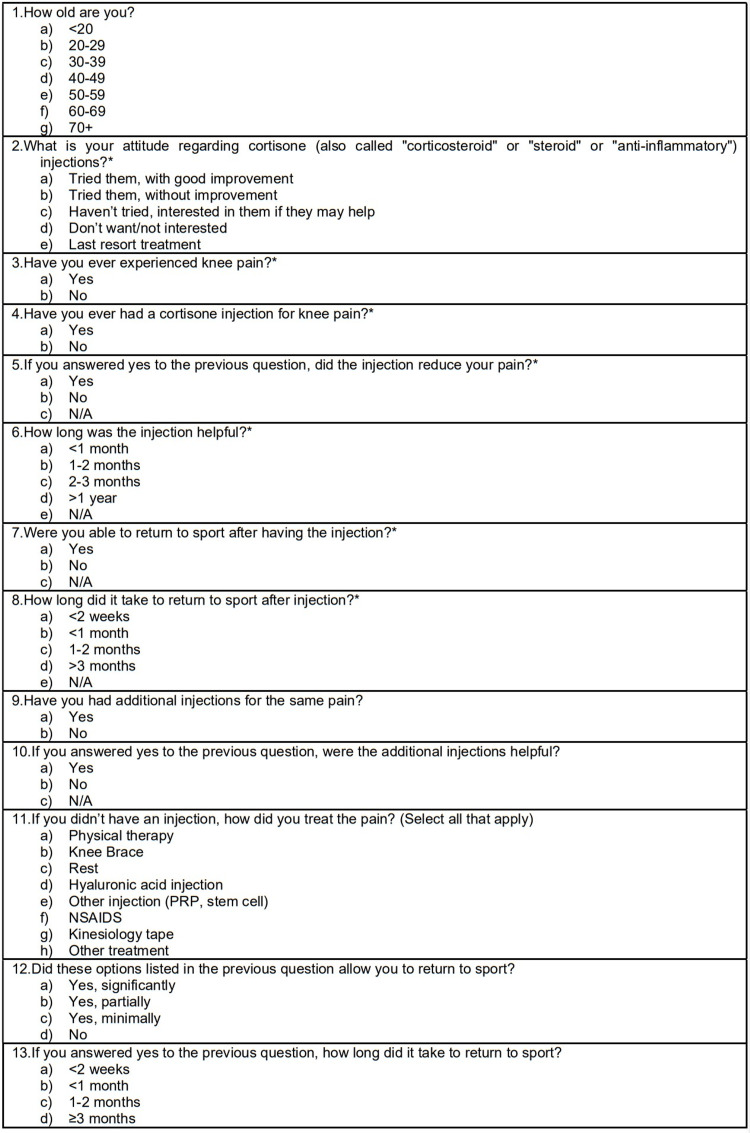
Survey questions administered. Questions with an asterisk (*) were required upon completion of the survey NSAIDS - non-steroidal anti-inflammatory medications, RPR - rapid plasma reagin

The 13-question electronic survey was posted to three social media internet platforms (Facebook, Slowtwitch, Reddit). The posting locations on these platforms were specific to triathlon groups (e.g., Women for Tri), triathlon teams (e.g., Team Zoot), and specific Ironman competition events (e.g., Ironman World Championship 2019, Ironman Texas, etc.). We targeted responses from at least 100 triathletes. This anonymous and voluntary survey provided informed consent but did not require formal written consent; subjects who did not click to agree to the informed consent did not fill out the survey. The survey was active for two weeks. Responses were completely anonymous, with no personal identifying information other than age range. All questions asked in the questionnaire are included in Figure 2.

Statistical analysis 

For analysis of the average age of individuals using cortisone injections versus alternative treatments, we calculated a weighted average using median age (of age range) and the number of people (in that age range); the average age was rounded to the nearest whole number. Data was compiled, and descriptive analysis was performed using Microsoft Excel version 16 (Microsoft; Redmond, Washington). Fisher's Exact test was performed using Prism 8 (GraphPad; San Diego, California) for evaluation of the relationship between injection and time to return to sport. For analysis of time to return to sport, longitudinal outcome data (<2 weeks, <1 month, 2-3 months, and >3 months) was condensed to <1 month and ≥1 month. An alpha level of 0.05 was used, with a p-value of <0.05 considered significant. 

## Results

**Figure 3:**
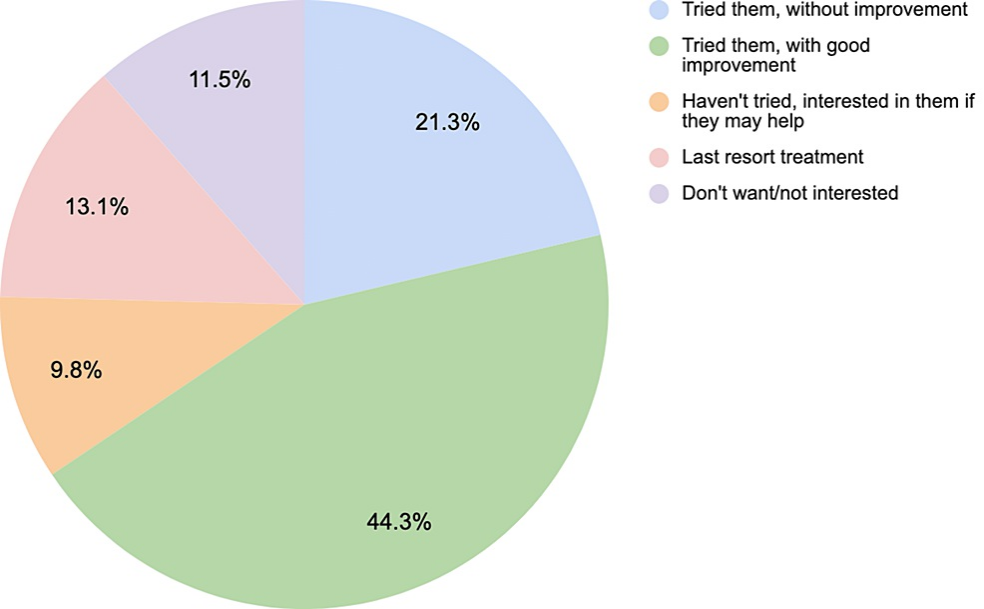
Respondent attitudes about cortisone injections *Responses from 61 participants

The study was conducted in 2020 during the initial phase of the COVID-19 pandemic, which may have led to lower response rates. There were 61 total responses to the survey. The survey was posted on social media sites, and participation was voluntary. Completion of core survey components was required prior to submition. Most respondents (n=59, 96.7%) had experienced knee pain. The mode age range was 50-59 years old (24.6%), and the age range with the lowest representation was <20 years old (1.8%); four people declined to enter their age (Table 1). The most popular attitude regarding cortisone injections was that people had "tried them, with good improvement" (44.3%). The least popular attitude regarding cortisone injections was "haven't tried them, interested in them if they may help" (9.8%). See Figure 3 for respondent attitudes toward cortisone injections. 

**Table 1:** Age distribution of respondents *Values reported as n (%); total respondents of age range n=57 (4 did not specify age range)

Age range						
<20	20-29	30-39	40-49	50-59	60-69	70+
1 (1.8%)	8 (14%)	11 (19.3%)	12 (21.1%)	14 (24.6%)	9 (15.8%)	2 (3.5%)

Sixty-four percent (64.4%, n=38) of respondents had received a cortisone injection for knee pain, and 73.7% (n=28, Table 2) experienced improved pain ranging from <1 month to >1 year. The average age of those receiving cortisone injections was 51 years. Of those who had a cortisone injection, 81.6% (n=31) were able to return to sport, the majority of whom (66.7%, n=20) returned in less than two weeks (Table 3). Most recipients found the injection helpful for ≥2 months (2-3 months (28.6%, n=8) or >1 year (28.6%, n=8)). Of the respondents who found the injection helpful for >1 year, 50% (n=4) had received multiple injections (Table 2). More than half (52.6%, n=20) of all respondents who received a cortisone injection for knee pain received an additional injection for the same pain, and all (100%) of those people found the additional injection helpful. 

**Table 2:** Reported length of time cortisone injection helpful *Values reported as n (%): n=38 received injection for knee pain, n=28 noted improvement status post-injection; 50% (n=4) of respondents with improvement >1 year had received multiple injections

Subjective duration of effectiveness			
<1 month	1-2 months	2-3 months	>1 year
7 (25.0%)	5 (17.9%)	8 (28.6%)	8 (28.6%)

**Table 3:** Reported length of time to return to sport after cortisone injection *Values reported as n (%); n=38 received injection for knee pain (n=28 positive improvement, n=10 no improvement), n=31 returned to sport (n=1 returned to sport, did not report time to return)

Time to return to sport			
<2 weeks	<1 month	1-2 months	>3 months
20 (66.7%)	5 (16.7%)	4 (13.3%)	1(3.3%)

Of the 96.7% that reported knee pain, 34% (n=20) utilized alternative treatment rather than cortisone (Table 4). The average age of those seeking alternative treatment was 36 years old. The most popular alternative treatments were physical therapy (65.0%, n=13), rest (55.0%, n=11), and use of a knee brace (35%, n=7) or other treatment (35%, n=7) (Table 4). Approximately 90% of those who used these alternative treatments reported that "yes, significantly" they were able to return to sport; approximately 10% were "yes, partially" able to return to sport. Using alternative treatment methods, most could return to sport within one month (<2 weeks: 42.1%, n=8; <1 month: 31.6%, n=6) (Table 5). 

**Table 4:** Reported alternative treatments used by type. *PT -physical therapy, brace - knee brace, kinesio tape - kinesiology tape, HAI - hyaluronic acid injection, NSAIDS - non-steroidal anti-inflammatory drugs; n=20 triathletes with knee pain using alternative treatments; data reported as n (%/alternative treatment respondents). # treatments greater than # respondents as people used multiple alternative therapies

Alternative treatment types							
PT	Brace	Rest	Kinesio tape	HAI	Other injection	NSAIDS	Other treatment
13 (65%)	7 (35.0%)	11 (55.0%)	5 (25.0%)	0 (0.0%)	1 (5.0%)	5 (25.0%)	7 (35.0%)

**Table 5:** Reported length of time to return to sport using alternative treatment methods *Data reported as n (%); n=20 used alternative treatments for knee pain, n=1 did not specify time to return to sport

Time to return to sport			
<2 weeks	<1 month	1-2 months	>3 months
8 (42.1%)	6 (31.6%)	3 (15.8%)	2 (10.5%)

**Figure 4:**
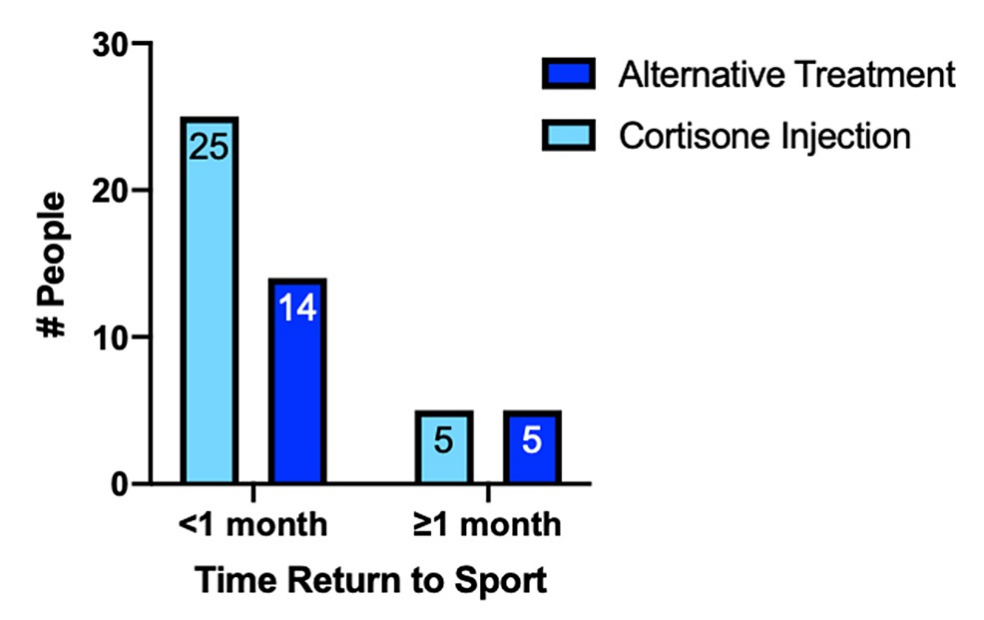
Time to return to sport using cortisone injections versus alternative treatments *Return was grouped as <1 month and ≥1 month. Bars represent the number of triathletes who returned to sport within that time period. For those returning <1 month, the majority were those who had received a cortisone injection (n=25, 64.1%). Return to sport <1 month did not differ significantly for cortisone injection versus alternative treatments (p=0.480, OR: 1.786, CI:0.448-7.09)

Though statistical analysis identified an 80% higher likelihood of returning to sport within a month using corticosteroid injections versus other treatment methods, the association was not significant. (OR=1.786, p=0.480, 95% CI:0.448-7.09) (Figure 4). 

## Discussion

Corticosteroid injections are controversial due to questions of efficacy and concerns regarding side effects [9], and therefore lack clear guidelines in the general athletic population [10]. Nonetheless, their potent anti-inflammatory pharmacologic effects have led to extensive use in the management of many athlete populations [4,11]. To our knowledge, this is the first study to examine corticosteroid injection use for knee pain, specifically in the triathlete population. These data suggest that triathletes may have a more positive attitude about corticosteroid injections and their utilization. These data further suggest that triathletes may use these injections with subjectively satisfactory results. Triathletes who tried corticosteroid injections tended to be older compared to those who used alternative treatments. We were unable to examine the reason behind this or if alternative treatments were trialed prior to corticosteroid injections. Unfortunately, there is no literature to date that examines corticosteroid use in triathletes, much less in older triathletes. One possible hypothesis behind elderly triathletes using corticosteroid injections may be related to the degenerative changes in the knee (i.e., osteoarthritis (OA)), which often develops with age; thus, the older population may be more prone to seek injections for relief of OA-related pain and swelling [12,13]. We point this out in highlighting the need for further research to be done to elucidate age-specific trends regarding the use of corticosteroid injections in triathletes. 

The majority of triathletes who received cortisone injections were able to return to sport within two weeks. Notably, there was an 80% higher odds of returning to sport within one month for those using corticosteroid injections compared to those who used alternative treatments. However, this relationship was not significant. Potential reasons for differences in effect and time to return to sport may also be explained by the practitioner injection technique and/or medical indication, as there are numerous methods for injection and varying knee diagnoses at the time of injection [5,14]. Further research with larger sample sizes, injection technique, and indication is needed to definitively assess if there is an association between corticosteroid injection and quicker return to sport compared to alternative treatments. 

Many triathletes found joint injections to be helpful both short-term (two to three months) and long-term (one year). Half (50%) of people who reported long-term (more than one year) relief had received multiple injections. We propose that the longer duration of effect may be due to the additive effect of multiple injections rather than long-term relief from one injection. However, this comparison would have to be analyzed in future studies. Our findings are supported by the fact that corticosteroids are generally accepted to have short-term efficacy for up to three months in the general athletic population [7], which aligns with our findings in triathletes. While we acknowledge that the general athletic population differs from the triathlete population, there are no existing studies on corticosteroid use in triathletes to facilitate comparing our results to findings in the existing literature. 

The tendency to place an inflammatory label ("-itis") on sports-induced pain has promoted the use of anti-inflammatory treatments among athletes [14]. However, given the relatively short duration of effects for most respondents (as well as the tendency of many individuals to receive multiple injections), our results suggest that corticosteroids may not be effective as a sole treatment in the long-term management of knee pain in triathletes. This finding agrees with prior recommendations on the role of corticosteroids in sports-related injuries [14], which recommend a multi-disciplinary, combined approach including approaches such as physical therapy/rehabilitation, appropriate activity modification, eliminating training errors, maximizing symmetry and biomechanics, development of correct technique, and possible well-timed surgical intervention as clinically indicated. 

The triathletes in our study who did not receive cortisone injections did end up using numerous alternate methods. We propose that this so-called "kitchen sink" approach may be due to the paucity of quality clinical evidence regarding these alternative treatment modalities and thus, the unknown efficacy of many of these treatments. Ultimately, the optimal combination of rehabilitation methods in triathletes remains unknown and is likely unique to each individual and specific to the differing characteristics of each injury. Further research is needed to establish which combination of approaches best complements the positive effects of corticosteroid injections under different circumstances. Alternatives to corticosteroid injection, such as platelet-rich plasma injection, should also be evaluated.

Although corticosteroid injections are used with the goal of ameliorating symptoms and facilitating the return to sport in the short term, they are not without risk of potential long-term complications. These risks include articular cartilage damage, decreased bone mass, fascial and tendon rupture, and overall joint degeneration [4,7]. These side effects are both important and relevant to the triathlete population as endurance sports have long-term risks inherent to the nature of the sport itself, such as decreased bone mass [15], tendon injuries [16], possible joint degeneration and arthritis progression [6,17,18]. These "side effects of the sport" are very similar to the aforementioned side effects of corticosteroid injections [13]. Therefore, triathletes may be at unique risk if they undergo corticosteroid injections. Additionally, recently there have been renewed concerns about joint disease progression with repeated corticosteroid injections [19], with some studies suggesting the local anesthesia given with injection may also contribute to a chondrotoxicity effect [20,21]. Unfortunately, precise estimates on complication rates following corticosteroid injection in the treatment of athletic injuries, much fewer injuries related to triathletes, do not yet exist in the medical literature [4]. However, because multiple corticosteroid injections may pose potential risks, this study helps to illustrate how prevalent cortisone injection use is in triathletes to better inform medical providers treating these athletes and counseling them on the risks. Finally, as the average age of corticosteroid use was older in our study, there are likely a combination of risk factors in our study participants (including progressive degenerative change inherent to both advancing age and the repetitive stress of the sport, which may be further increased by the additional corticosteroid side effect risks). Therefore, older adult triathletes considering cortisone injections should be counseled on potential compounded risks. 

It is recommended that corticosteroid injections shouldn't be given immediately after the injury or immediately prior to sports participation [22-24]. The consensus statement by the International Olympic Committee [22] indicates no same-day return to play after injection, and the World Anti-Doping Agency (WADA) in 2017 prohibited injections <72 hours before competition [22] due to the risks of acute weakening of the tendons and muscles [23]. Corticosteroid-induced pain inhibition may also make athletes more vulnerable to tissue overload and failure [14]. These side effects can persist for up to three months [13]. In our study, most triathletes returned to sport within two weeks, potentially putting them at risk based on current recommendations for the general athlete population. However, it must be understood that current recommendations and past research on runners, or on athletes in general, may not be generalized to what we might expect in triathletes. In addition, the therapeutic effect of corticosteroids may also be partially negated by vigorous activity immediately before or shortly after injection [22]. This valuable point should also be conveyed to triathletes who are more concerned with return to sport. 

Given that triathletes are already at risk for overuse injuries, we recommend counseling triathletes on the timing of injections, duration of side effects, and potential risks. Corticosteroid injections certainly ameliorate excessive inflammation. However, anti-inflammatory therapy may only succeed if the athlete has been educated on proper expectations and responsibilities [14]. Given the known potential risks, possible side effects, and our findings of an insignificant association with faster return to sport, other alternatives should also be considered when counseling triathletes on corticosteroid injections. 

Limitations

We recognize the limits inherent to our study. A clear limitation is the observational nature of the study, as no interventions could take place during the initial phase of the COVID-19 pandemic. Further, the smaller sample size of this study limited our statistical analysis, such as the ability to perform a multinomial logistic regression using corticosteroid injection use for knee pain as a predictor for return to sport. It's also possible that the smaller sample size led to statistically insignificant associations when truly an association existed. We recognize the potential for recall bias inherent in a retrospective study. To decrease inaccuracy associated with retrospective recall, we used categorical variables to measure longitudinal outcomes as we believe people tend to estimate time frames, and thus, there would be more inaccuracy in asking for precise recall. We did not ask about chronicity nor medical diagnoses confirming the etiology of knee pain (e.g., patellofemoral pain syndrome, OA, etc.). Due to limitations of surveys, such as recall bias, we could not reliably measure specific details regarding the procedure, such as who performed the injections or the anatomical approach of the injection. While these are important questions for future research, they are outside of the scope of our study, which was focused on obtaining the first data on the effectiveness of corticosteroid injections in triathletes. Finally, the degenerative changes that occur with age may explain the higher use among older triathletes. Future randomized controlled trials with a larger sample size and a control group of individuals who had no treatment for knee pain may further clarify if the higher odds in our study represent a true change in return to sport timing with corticosteroid injections.

## Conclusions

This is the first study to examine corticosteroid use in triathletes and provide return to sports data. Our results suggest that corticosteroid injections are perceived positively and are commonplace among triathletes. While our study suggested a higher odds of returning to sport earlier with corticosteroid injection use compared to alternative methods, the association was not significant. Future investigations should further delineate the role of cortisone injections in return to sports and long-term knee pain treatment. In addition, triathletes should be counseled on expectations and management of injection timing given the potential side effects of corticosteroid injections, especially since triathletes may have additional musculoskeletal risk due to age and the demanding nature of the sport. 
